# Helping older people get the eye care they need

**Published:** 2008-06

**Authors:** Henry Nkumbe

**Affiliations:** CBM Consultant Ophthalmologist, FLM-SALFA Eye Hospital, PO Box 244, Antsirabe 110, Madagascar.

**Figure:**
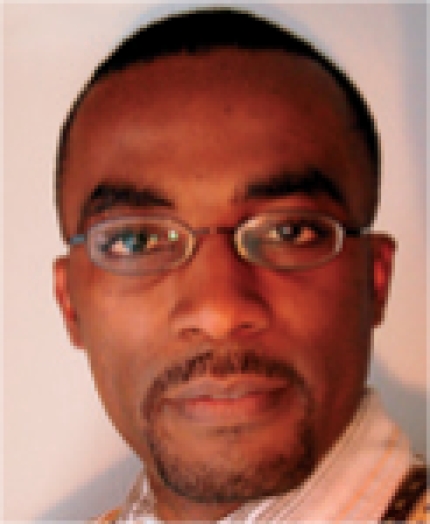


Despite being more affected by visual impairment and blindness than any other population group, older people are the group least likely to seek help when faced with eye problems or a deterioration of their vision. Even in the Kilimanjaro region in Tanzania, one of the few regions in Africa to have an excellent community eye care programme, it is estimated that only one in three older people with cataract actually receives an operation.

This article looks at some of the most important reasons that older people don't get the eye care they need.

## Cost of eye care

Worldwide, particularly in low- and middle-income countries, older people tend to be the poorest members of society.^1^ As a result, the cost of eye care services is a major issue, especially when people have to pay for their own health care.^2^

There are direct and indirect costs to eye care. In one region in Madagascar, for example, we recently discovered that indirect costs such as transportation and meals for older people and their caretaker(s) can amount to more than three times the price of a cataract operation or ten times the price of presbyopic spectacles. Such indirect, or hidden, costs (which can even include bribes) tend to discourage many older people from seeking treatment.

Barriers related to direct cost can be reduced by introducing a tiered pricing system which allows patients to pay according to their ability. One hospital the author worked in had such a system for their consultation fees: US $0 for patients who were too poor to pay, US $1 for regular patients, and US $20 for ‘fast-track’ patients (who paid extra for special waiting areas and shorter waiting times). Such a system should be clearly formulated and transparent to users and should make provision for people who cannot afford to pay for the services.

To help with indirect costs, a fee system could be set up where the fee for treatment includes transportation, meals for the patient and his or her caretaker, medicines, and so on. This eliminates any hidden costs and allows patients and their families to budget sensibly and in advance.^3^

Occasionally, patients give 'lack of money' as a reason for refusing an operation when there are deeper, more complex reasons that they may not wish to divulge. Proper counselling and repeat visits by field workers, outreach workers, or community workers can explore these reasons and often help the patient make an informed decision about surgery.

**Figure:**
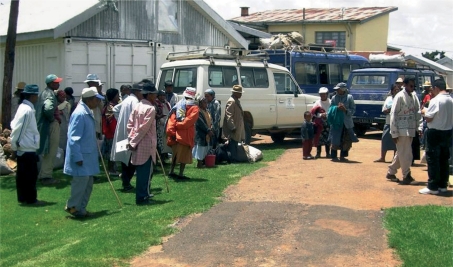
Organised transport to an eye clinic. These older persons were diagnosed with cataract in a remote district. MADAGASCAR

## Fear of eye surgery

Fear of eye surgery is universal, even among eye care workers - which should help eye care workers be more sympathetic to the fears of others.

The attitude and skill of an eye care team will determine how successful it is at alleviating fear in potential patients. However, a well-trained social worker, counsellor, or patient advisor may be more successful than an eye surgeon at calming an older person's fear and winning their confidence. Older people who have successfully received treatment may also be trained to promote a service to their peers.

## Difficulty getting to eye care facilities

Most eye care facilities are located in urban areas, whereas older people usually live in rural areas.

Eye care programmes need to include comprehensive outreach services as one of their strategies. Such services should locate older people in need of eye care in the community; they should not be limited to screening for cataract and should look for other problems which may lead to visual impairment. These services could also provide refractive services, including the sale of ready-made spectacles.

Where they exist, community-based services can provide a link with a more distant eye care facility. Community-based rehabilitation workers can usually help with the following:

- identifcation and referral of older people with visual problems
- follow-up in the community
- low vision and rehabilitation services for those who may not benefit from surgery.

## Poor communication

Another major barrier is lack of awareness about treatable eye conditions, about where help can be sought, and about the real cost involved in eye treatment.^2^^,^^3^ This lack of awareness is compounded by a lower level of literacy among older people in Africa.

In order to overcome this barrier, it is important to work closely with the community and ensure that two-way communication takes place; eye care workers should not only talk to older people, but also listen to them. This builds trust and helps health workers understand what the real issues are.

It is also important to remember that the decision to seek medical help can be quite complex. In many low- and middle-income countries, the final decision is not made by the patient, but by the family (children, grandchildren, husband or wife, etc.) and peers (see article on page 31). Eye care workers, where possible, should help these decision makers to understand the socioeconomic benefits of good eyesight for older adults (see article on page 24).^5^

Spend some time finding the most efficient channel for the dissemination of information; this may depend on the specific situation. For example, a recent study in Tanzania suggested that churches with were the most effective channel in a predominantly Christian community.^6^ Our preliminary observations in Madagascar suggest that working with local community leaders and pension pay points may also be very effective.

Help the Aged, a UK charity which works in partnership with HelpAge International, suggests the following for improving awareness of eye care among older people:

- Ensure eye clinics are well promoted within a local area, bearing in mind language and communication channels: a leaflet may not always be appropriate if there are low literacy levels. Some alternatives are radio announcements or advertisements, and using community health workers to spread the message.
- Pay special attention to explaining any costs; also explain what assistance is available to help older people meet those costs.
- Where treatment is free, ensure this is well understood and that any indirect costs are explained.
- Forge long-term links between non-governmental organisations and other representative groups working with older people.

## Gender inequalities

Many studies have shown that, although women account for more than 60 per cent of people living with visual impairment, they are less likely to seek help than men. In some areas, this barrier is even more significant for older women, especially those who are widowed or childless, as hey have less social support.

Proper counselling and organised transportation seem to be extremely important in improving access to eye care for women. In addition, special education programmes with women's groups could help increase awareness about eye health. It is also important that men, who often have the final say, understand that the rights of women to good vision are as valid as those of men.^7^

## Insufficient collaboration among professionals who care for older people

In addition to eye problems, older people usually have other age-related health problems, such as hearing impairment, arthritis, cardiovascular disorders, and diabetes (see article on page 31).

The disability caused by such disorders could make some older people reluctant to visit health facilities. When they do visit, the health team very often doesn't check their eyes, even though there might be an eye department in the health facility and these older people may have visual impairment or a sight-threatening condition.

The general health of older people is not a topic often included in the training curricula of eye personnel. This, coupled with overspecialisation, results in many eye care workers feeling overwhelmed by the health problems of older people when these are not related to the eye.

It is necessary to develop a team approach. In large hospitals, the eye department should work closely with other relevant specialties. In stand-alone eye units, experienced physicians, geriatricians, paramedics, and community nurses need to be identified who can help with the management of health problems faced by older people.

**Figure:**
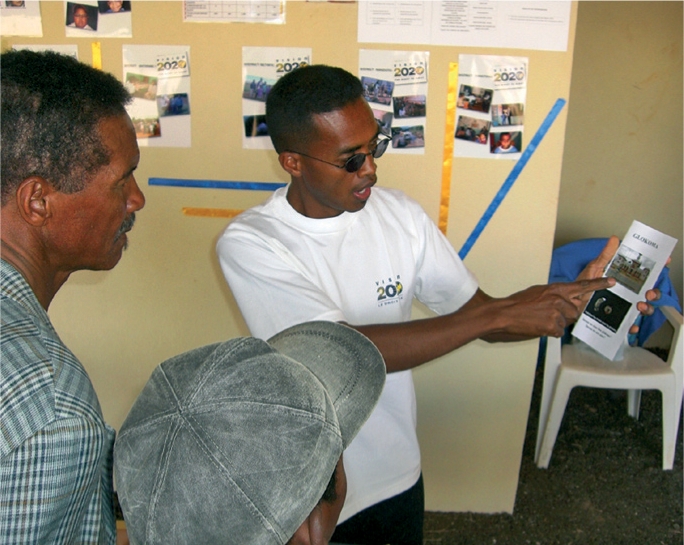
A Community eye health field assistant uses a flyer to counsel an older person about glaucoma. MADAGASCAR

## Living in a nursing home

The prevalence of visual impairment among older people in care institutions is much higher than in similar groups in the general population. The author recently found nursing home residents blind from cataract in Madagascar, although the home was a ten-minute drive from a reputable eye unit offering cataract surgery.

This problem can be solved by working closely with nursing homes and other care institutions, where they exist, and arranging regular eye examinations that include low vision services (which should be offered at these institutions, where possible).

## Not perceiving the need for eye care

Many older people consider poor vision to be normal in later years. In some places, it even earns them respect and certain privileges. In some Muslim communities, for instance, blind older people are responsible for making the call to prayer at the mosque or for reciting the Qur'an during public events. Faced with other pressing needs, family members may also give less priority to the health of their older relatives.

Proper information and counselling, as well as public information programmes, may be needed to overcome this barrier.

## HIV and AIDS

HIV and AIDS have resulted in a ‘missing’ generation in many communities in sub-Saharan Africa. In severely affected areas, it is estimated that nearly half of older people have either lost their children or have to take care of their children who are sick; they often have to foster their grandchildren as well. Not only is the financial support they might have expected from their children gone, but the little money they might have left is also used for the welfare of their children and grandchildren. Their own health becomes less of a priority and they become physically and emotionally drained.

Eye care programmes need to partner with programmes for people living with HIV and AIDS, especially home-based care programmes. They need to advocate for the health of older people who act as caretakers. Caretakers need recognition, information, and financial support, not just for the sake of their children and grandchildren, but also for the sake of their own health.

## Going forward

The global elimination of avoidable blindness by 2020 will depend largely on how well we address the needs of older people and the barriers to eye care they face worldwide.

Understanding and overcoming these barriers is an ongoing process. It may be helpful to put a tracking system in place to monitor people who don't come for treatment after they have been identified in the community. If possible, return visits should be made to find out what the problems are and to work with older people and their families to solve them.

**How to ensure a clinic is age-friendly**

Like all other service users, older people must be treated with dignity and respect; they should not feel embarrassed to ask for any extra help they may need.

**Physical considerations**

It is important to ensure that older people are able to use the building independently, where possible.

- When designing a building, use ramps in addition to steps where possible and remove any obstacles which could make people trip over.
- Toilets should be spacious, with handrails.
- Rooms should be well lit and glare shoud be avoided.
- The wording on all signs in a building should be in mixed-case large print; the background should be in a contrasting colour.
- There should be plenty of seating available at reasonable height; this should not obstruct the areas where people are likely to walk. Seating should be in a contrasting colour to that of the floor.
- Treatment areas should maintain the privacy of a patient: ensure treatment take place in separate areas, screened off from other patients.

**Other considerations**

- Staff should be patient and take time to explain processes to older people without being patronising. They should listen carefully to any concerns. Where possible, staff should be given basic awareness training and be sensitive to the needs of older people with visual impairment.
- Older people often like to be informed in advance so that they can be ready and prepared. For example, ask them to bring lists of their current health conditions and drugs with them. As there is often a limit to the amount of time older patients can concentrate for, asking them to prepare in advance both speeds up the consultation (and improves its accuracy) and allows patients to be fresh for the important part of the consultation (what disease they have and what treatment they will need, etc.).
- It may be very helpful to use volunteers. They can let older people know where they are in the queue; this allows an older person to visit the toilet without being anxious about losing his or her place, for example. Volunteers can also get older patients ready and deliver them to the consultation room. This has the double advantage of not taking up the professional time of the nurse/ophthalmologist and of letting patients take their own time so that they do not arrive flustered, out of breath, or anxious.

With thanks to Help the Aged, www.helptheaged.org.uk
